# Diagnostic performance of contrast-enhanced ultrasound in traumatic solid organ injuries in children: a systematic review and meta-analysis

**DOI:** 10.1007/s00247-024-06127-9

**Published:** 2024-12-13

**Authors:** Payam Jannatdoust, Parya Valizadeh, Amir Hassankhani, Melika Amoukhteh, Delaram J. Ghadimi, Mahsa Heidari-Foroozan, Paniz Sabeghi, Paniz Adli, Jennifer H. Johnston, Pauravi S. Vasavada, Ali Gholamrezanezhad

**Affiliations:** 1 School of Medicine, Tehran University of Medical Sciences, Tehran, Iran; 2 Department of Radiology, Keck School of Medicine, University of Southern California (USC), 1441 Eastlake Ave Ste 2315, Los Angeles, CA 90089 USA; 3 Department of Radiology, Mayo Clinic, Rochester, MN USA; 4 School of Medicine, Shahid Beheshti University of Medical Sciences, Tehran, Iran; 5 Non-Communicable Diseases Research Center, Endocrinology and Metabolism Population Sciences Institute, Tehran University of Medical Sciences, Tehran, Iran; 6 Student Research Committee, School of Medicine, Shahid Beheshti University of Medical Sciences, Tehran, Iran; 7 College of Letters and Science, University of California, Berkeley, CA USA; 8 Department of Diagnostic and Interventional Imaging, McGovern Medical School, University of Texas Health Science Center at Houston, Houston, TX USA; 9 Department of Radiology, University Hospitals Case Medical Center, Case Western Reserve University School of Medicine, Cleveland, OH USA

**Keywords:** Abdominal injuries, Blunt abdominal trauma, Contrast-enhanced ultrasound, Diagnostic imaging, Pediatric trauma, Solid organ injuries

## Abstract

**Background:**

Blunt abdominal trauma (BAT) is a significant contributor to pediatric mortality, often causing liver and spleen injuries. Contrast-enhanced computed tomography (CT), the gold standard for diagnosing solid organ injury, poses radiation risks to children. Contrast-enhanced ultrasound (CEUS) may be a promising alternative imaging modality.

**Objectives:**

To evaluate the diagnostic utility of CEUS for detecting solid organ injuries following BAT in the pediatric population.

**Methods:**

A systematic review and meta-analysis were conducted through a thorough literature search in PubMed, Scopus, Web of Science, and Embase databases up to October 1, 2023. Diagnostic accuracy metrics were aggregated using a bivariate model, and subgroup meta-analysis compared CEUS accuracy across various organs.

**Results:**

Meta-analysis from four studies, including 364 pediatric patients, revealed a pooled sensitivity of 88.5% (95%CI 82.5–92.6%) and specificity of 98.5% (95%CI 94.9–99.6%), with an area under the curve of 96% (95%CI 88 – 99%). Splenic injuries showed higher sensitivity than liver injuries (*P*-value < 0.01), while kidney assessments demonstrated higher specificity (*P*-value < 0.05).

**Conclusion:**

This study highlights the diagnostic potential of CEUS for pediatric solid organ injuries caused by BAT. Further large-scale studies are needed due to the limited number and sample size of the included studies.

**Supplementary Information:**

The online version contains supplementary material available at 10.1007/s00247-024-06127-9.

## Introduction

Trauma is the leading cause of death among children worldwide, and blunt abdominal trauma (BAT) is a major cause of morbidity and mortality in this age group [1]. The spleen, followed by the liver, is the most commonly injured organ following BAT and poses a significant risk for life-threatening bleeding [1, 2]. The mortality rate for pediatric BAT is reported to be 10%, depending on the severity of the injury, concurrent injuries, and the effectiveness and timeliness of medical interventions [3].

Although there are limited studies assessing the performance of alternative methods focusing on the pediatric population, contrast-enhanced computed tomography (CE-CT) is considered the gold standard method for detecting solid organ injuries [4]. However, the reliance on CT scans for identifying traumatic injuries in children raises concerns regarding the potential risks of ionizing radiation exposure [5]. Although the cumulative risk of fatal malignancies from a single CT scan is very low, evidence suggests that CT imaging is frequently overused in pediatric trauma cases, and it is generally recommended to minimize this exposure by utilizing alternative modalities that do not involve ionizing radiation [6–8].

Ultrasound is widely recognized for its utility in trauma settings, offering rapid bedside evaluation using portable equipment through focused assessment with sonography for trauma (FAST) protocols. These protocols have high specificity for detecting free peritoneal fluid, helping to reduce unnecessary CT scans [9, 10]. However, beyond the scope of FAST, which is primarily a screening tool, ultrasound—including diagnostic ultrasound performed by radiologists—has limited value in detecting solid organ injuries when compared to CT [11–13].

As a novel ultrasound technique, contrast-enhanced ultrasound (CEUS) is effective in evaluating solid organ injuries, revealing trauma-induced lesions such as lacerations, hematomas, and active hemorrhages that may go undetected on standard diagnostic ultrasound [14–16]. This technique has demonstrated particular promise in assessing challenging anatomical regions, including the spleen, offering a safer alternative for point-of-care evaluations by eliminating ionizing radiation risks [17–19]. CEUS enables dynamic evaluation of extravasation patterns, an advantage over CT that contributes valuable information in trauma assessment and has demonstrated excellent safety profiles in both adults and children [17–19]. Emerging evidence suggests that CEUS may achieve diagnostic performance comparable to CE-CT, indicating its potential as a valuable tool in managing BAT [20, 21].

While a previous systematic review by Pegoraro et al. concluded that CEUS can be considered a safe and accurate imaging modality for pediatric BAT, to the best of our knowledge, no prior meta-analysis has quantitatively analyzed the diagnostic performance of CEUS in pediatric BAT [22]. In this meta-analysis, we aim to assess the overall diagnostic performance of CEUS for solid organ injuries in pediatric patients resulting from BAT.

## Methods

This systematic review adheres to the guidelines stipulated in the Preferred Reporting Items for Systematic Reviews and Meta-Analyses (PRISMA) statement [23]. On October 1, 2023, a comprehensive literature search was conducted across four major databases: PubMed, Scopus, Web of Science, and Embase. Tailored search terms were formulated for each database, encompassing (“contrast-enhanced ultrasonography” OR “contrast-enhanced ultrasound” OR “CEUS”) AND (“trauma*” OR “injur*”) AND (“pediatric*” OR “paediatric*” OR “child*” OR “neonat*” OR “infant*” OR “toddler*” OR “preschool” OR “pre-school” OR “juvenile” OR “young adult*”). Additionally, a comprehensive manual review of references within the included studies was conducted to ensure no relevant papers were inadvertently overlooked.

The assessment process involved a review of each article’s title, abstract, and/or full text. Two co-authors independently conducted this review, resolving any uncertainties or ambiguities through consultation with a senior co-author. Deduplication, screening, and data extraction were facilitated using the AutoLit platform, developed by Nested Knowledge in St. Paul, MN, USA.

All studies relevant to the topic of interest in patients < 18 years, reporting at least one of the following diagnostic accuracy measures, were eligible for inclusion: sensitivity, specificity, positive predictive value (PPV), negative predictive value (NPV), likelihood ratio (LR), diagnostic odds ratio (DOR), and area under the receiver operating characteristic curve (AUC). No restrictions were placed on publication date, country of origin, patient characteristics, reference standard type, or study design. Non-English literature, case reports, case series with fewer than five eligible patients, conference abstracts, editorial comments, and review articles were excluded from the study. Case reports and series with fewer than five cases were specifically excluded to avoid the potential for misleading results from a very limited and non-random sample.

The quality assessment of diagnostic accuracy studies-2 (QUADAS-2) tool was employed to evaluate the quality of included studies [24]. The four primary domains of the QUADAS-2 tool, namely patient selection, index test, reference standard, and flow and timing, underwent independent assessment for potential bias and concerns regarding applicability. Assessments for each domain were based on predefined criteria outlined in the tool, such as the representativeness of the study population, blinding of test results, and completeness of outcome data. Ratings of “low,” “high,” or “unclear” were assigned to each domain to ascertain the overall reliability of the evidence synthesis.

### Statistical analysis

The meta-analysis began by extracting true positives, true negatives, false positives, and false negatives from the selected studies. Our primary methodological framework was a bivariate random effects diagnostic test accuracy (DTA) model, as introduced by Reitsma et al. [25]. This approach facilitated the creation of summary receiver operating characteristic (SROC) curves using the bivariate meta-analysis data. Within these SROC plots, the size of the study-specific point estimates representation was determined by their proportional weight within a random effects univariate DOR model. The AUC and its confidence interval for each subgroup were computed using a 2,000-sample bootstrapping method based on the bivariate model [26].

The analysis unfolded in two phases. Initially, an “overall” meta-analysis pooled the diagnostic test accuracy data of CEUS across various reported solid organ injuries, incorporating results from all organs noted in each study. Subsequently, we conducted a subgroup meta-analysis hypothesizing that diagnostic effectiveness might differ across different organs. This phase focused on subgroups representing any single organ mentioned in at least three distinct studies and also involved a comparative evaluation of diagnostic performance across various organs.

The *I*^2^ metric, following Holling et al.’s methodology, was employed to assess heterogeneity [27]. A confidence interval for *I*^2^ exceeding 50% indicated significant heterogeneity, prompting additional sensitivity analyses via the DOR univariate meta-analysis to detect and reassess the effects of potential outliers.

The clinical relevance of our findings was evaluated using Fagan plots and likelihood ratio scattergrams. Positive likelihood ratios greater than 10 were interpreted as indicative of suitability for confirmation, while negative ratios under 0.1 suggested exclusion. Fagan nomograms were developed for pre-test probabilities of 25%, 50%, and 75%, derived from the bivariate model estimates, aligning with the recommendations of Zwinderman et al. [28].

To assess the presence of publication bias or small study effects, paired funnel plots for sensitivity and specificity were examined. Due to the limited number of studies included, Egger’s regression test was not applied, and asymmetry in the funnel plots was assessed visually.

All statistical analyses were conducted using R software (version 4.2.1, R Foundation for Statistical Computing, Vienna, Austria), utilizing key packages such as “Mada,” “dmetatools,” “Metafor,” and “meta” [26, 29, 30]. A *P*-value lower than 0.05 was considered significant throughout the analysis.

## Results

### Article screening and selection process

**Fig. 1:**
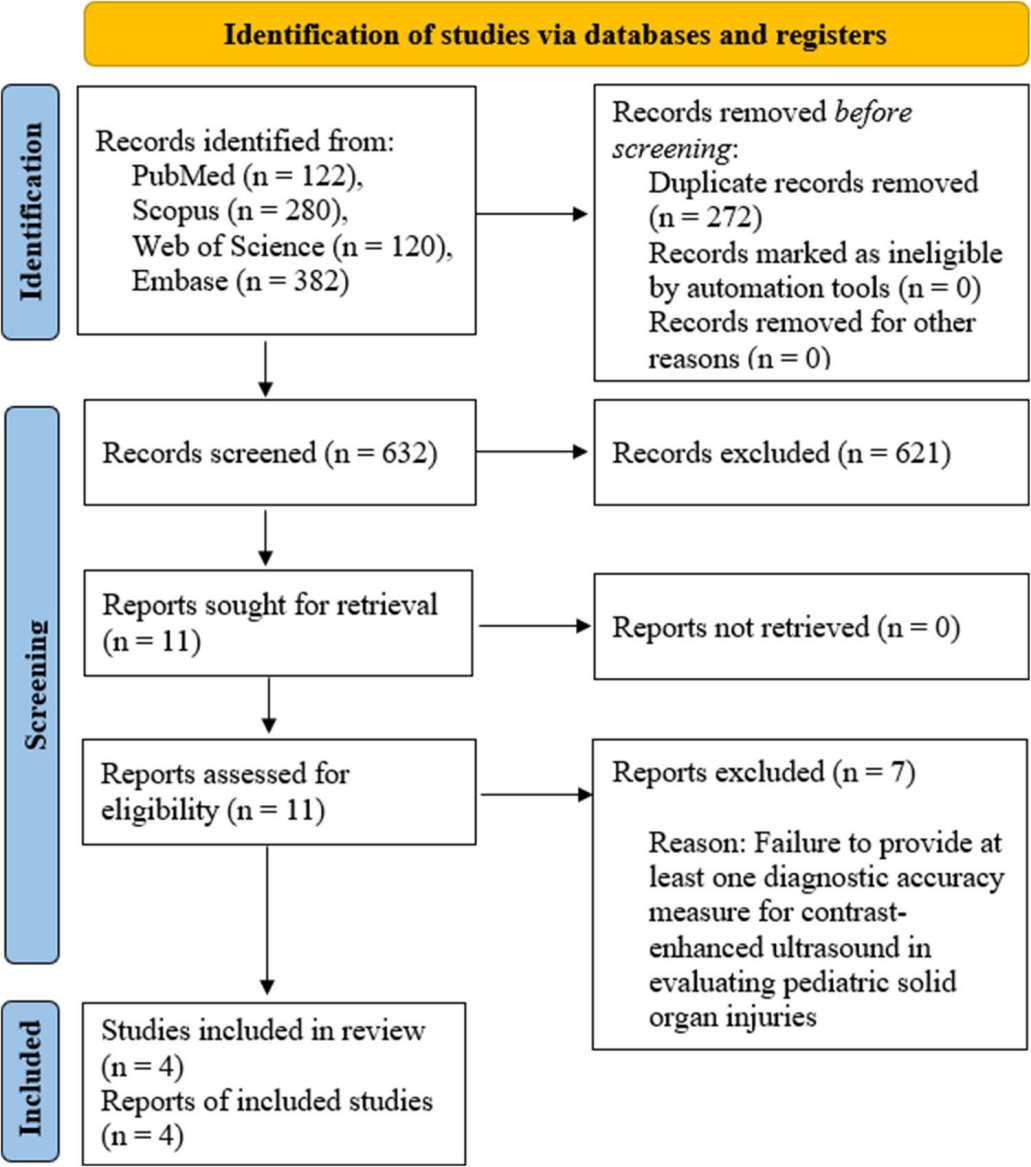
PRISMA flow diagram showing the review process. PRISMA, Preferred Reporting Items for Systematic Reviews and Meta-Analyses

A systematic literature search utilizing a predefined strategy identified 904 articles. After removing duplicates, 632 articles underwent screening based on title and abstract, resulting in the exclusion of 621. The full text of the remaining 11 articles underwent meticulous review. Seven articles were excluded after thorough examination because they did not report at least one diagnostic accuracy measure for CEUS in evaluating pediatric solid organ injuries. Ultimately, four articles meeting the inclusion criteria were identified and included. These studies provided sufficient data for constructing 2 × 2 tables, facilitating the DTA meta-analysis. The screening process and eligibility criteria adhered to PRISMA guidelines, with a flow diagram presented in Fig. 1.

### Study characteristics

This review incorporates findings from four studies assessing the diagnostic efficacy of CEUS in identifying solid organ injuries among 364 pediatric patients. The studies included in this review were geographically diverse: two were conducted in Italy, one in the USA, and one in Saudi Arabia [20, 31–33]. All studies utilized a cohort approach, two prospective and two retrospective.

The diagnostic performance of CEUS was specifically reported for various solid organs. Injuries to the spleen and liver were examined in all studies, while kidney injuries were assessed in three studies [20, 32, 33]. Pancreatic injuries were reported in two studies, and adrenal gland injury was reported in only one study, preventing us from performing subgroup analysis for these lesions [32, 33].

All studies used CT scans as the reference standard, specifically mentioning contrast-enhanced CT imaging in all but Armstrong et al.’s study [33]. Importantly, CEUS was performed by radiologists at the point of care (POC) in all included studies. However, the studies demonstrated discrepancies in sample inclusion criteria, utilized ultrasound equipment and techniques, and the definitions of solid organ injuries. Detailed information on these aspects is provided in Table 1. Noteworthy is that the study by Menichini et al. only included patients with abnormal findings on unenhanced diagnostic ultrasound [20].

**Table 1:** Characteristics of the included studies

Author, year	Country	Study design	Age	Number of patients (F:M)	Inclusion criteria and type of trauma	Exclusion criteria	Studied organs, with types and numbers of injuries in each organ (based on reference tests)	Ultrasound equipment/probe type/frequency/contrast material	Performer/setting	Criteria to identify injuries	Reference test	Sensitivity	Specificity
Armstrong, 2017	USA	Prospective single center	Mean 13.3 Range (7–18)	18 (6:12)	Hemodynamically stable	Known cardiac or pulmonary injury/abnormalities, albumin or blood product sensitivity, pregnancy, inability to provide assent, or inability to roll over for the exam	Kidney: 4 Liver: 3 Pancreas: 0 Spleen: 14	GE LOGIQ E9/curved probe/1–5 MHz/Optison™ (suspension of microspheres of human serum albumin with perflutren)	Pediatric attending radiologists blinded to CT results/POC	Persistently hypoechoic or anechoic foci that were stable in appearance throughout the examination and located within the parenchyma in the case of a laceration or contusion or in a subcapsular location in the case of a hematoma or urinoma	CT scan	Kidney: 87.5% Liver: 66.67% Pancreas: NA Spleen: 89.3%	Kidney: 100% Liver: 100% Pancreas: 100% Spleen: 100%
Zakaria, 2023	Saudi Arabia	Retrospective multi−center	Mean±SD 10.5±2.2 Range (1–16)	246 (96:150)	Hemodynamically stable blunt abdominal trauma	Blunt trauma with extra−abdominal major injuries, hemodynamically unstable after adequate resuscitation, early or subsequent generalized peritonitis	Liver: 93 Spleen: 168	Siemens Sonoline Elegra/linear and curved probe/2.5–7.5 MHz/LUMASON® (sulfur hexafluoride lipid−type A microspheres)	Two independent attending radiologists/POC	Not reported	CE CT scan	Liver: 75.6% Spleen: 89.5%	Liver: 95.2 Spleen: 94.6%
Menichini, 2015	Italy	Retrospective single center	Mean±SD 8.7±2.8 Range (0–16)	73 (22:51)	Hemodynamically stable patients with minor blunt abdominal trauma and at least one positive finding in ultrasound	Adulthood, hemodynamical instability, history of major trauma, negative US findings	Kidney: 20 Liver: 21 Spleen: 26	Siemens Acuson Sequoia 512/linear and curved probe/frequency not specified/Sonovue (second−generation blood pool contrast agent)	Radiologist blinded to the results of CT/POC	The presence of a hypoechoic area persisted unchanged during all the acquisition phases, with a subcapsular distribution in the case of hematoma or a parenchymal localization in the case of lacerations. The presence of intralesional hyperechoic spots was interpreted as a sign of active bleeding	CE CT scan	Kidney: 100% Liver: 100% Spleen: 100%	Kidney: 100% Liver: 100% Spleen: 100%
Valentino, 2008	Italy	Prospective single center	Mean±SD 8.9±2.8 Range (4–13)	27 (8:19)	Hemodynamically stable, moderate, or severe injuries, according to the Abbreviated Injury Scale	Hemoperitoneum, unstable vital signs, minor injury according to the Abbreviated Injury Scale and negative US findings, normal hematocrit levels, and normal hepatic and pancreatic enzyme levels	Kidney: 1 Liver: 4 Pancreas: 1 Spleen: 7 Adrenal: 1	Philips ATL HDI 5000/curved probe/2–5 MHz/Sonovue	Radiologists blinded to the results of CT/POC	Organ injuries appeared as strongly hypoechoic areas against the homogeneous echogenicity of the parenchyma with or without interruption of the anatomic profile. Microbubbles within the lesion were identified as active bleeding	CE CT scan	Kidney: 100% Liver: 100% Pancreas: 100% Spleen: 100% Adrenal: 0%	Kidney: 100% Liver: 100% Pancreas: 100% Spleen: 100% Adrenal: 100%

*CE*, contrast enhanced; *CT*, computed tomography; *F*, female; *GE*, General Electric; *M*, male; *MHz*, mega−hertz; *POC*, point of care; *SD*, standard deviation; *US*, ultrasound; *USA*, United States of America

### Quality assessment

The methodological quality of the included studies, as evaluated using the QUADAS-2 tool, is detailed in Table 2. This assessment indicates that the studies generally maintain acceptable methodological quality across all evaluated domains, with no major concerns identified. However, ambiguities were noted in the patient selection methods of three studies [20, 31, 33]. Additionally, the blinding of interpreters of the reference test (CT) to the results of CEUS, as well as the blinding of CEUS interpreters to the results of the reference test, was not explicitly addressed in some of the studies [31, 33].

**Table 2:** Results of risk of bias assessment using the QUADAS-2 tool

Author, Year	Q1	Q2	Q3	Q4	Q5	Q6	Q7	Q8	Q9	Q10	Q11	Q12	Q13	Q14	Q15	Q16	Q17
Armstrong, 2017	Unclear	Yes	Unclear	Unclear	No	Yes	Yes	Low Risk	No	Yes	Unclear	Low Risk	No	No	Yes	Yes	Low Risk
Zakaria, 2023	Unclear	Yes	Unclear	Unclear	No	Unclear	Yes	Unclear	No	Yes	Unclear	Low Risk	No	Yes	Yes	Yes	Low Risk
Menichini, 2015	Yes	Yes	Unclear	Low Risk	No	Yes	Yes	Low Risk	No	Yes	Yes	Low Risk	Yes	Yes	Yes	Yes	Low Risk
Valentino, 2008	Yes	Yes	Yes	Low Risk	No	Yes	Yes	Low Risk	No	Yes	Yes	Low Risk	No	Yes	Yes	Yes	Low Risk

Q1. Was a consecutive or random sample of patients enrolled?

Q2. Was a case-control design avoided?

Q3. Did the study avoid inappropriate exclusions?

Q4. Could the selection of patients have introduced bias?

Q5. Are there concerns that the included patients and setting do not match the review question?

Q6. Were the index test results interpreted without knowledge of the results of the reference standard?

Q7. If a threshold was used, was it pre-specified?

Q8. Could the conduct or interpretation of the index test have introduced bias?

Q9. Are there concerns that the index test, its conduct, or its interpretation differ from the review question?

Q10. Is the reference standard likely to correctly classify the target condition?

Q11. Were the reference standard results interpreted without knowledge of the results of the index tests?

Q12. Could the reference standard, its conduct, or its interpretation have introduced bias?

Q13. Are there concerns that the target condition, as defined by the reference standard, does not match the question?

Q14. Was there an appropriate interval between the index test and reference standard?

Q15. Did all patients receive the same reference standard?

Q16. Were all patients included in the analysis?

Q17. Could the patient flow have introduced bias?

### Publication bias

**Fig. 2:**
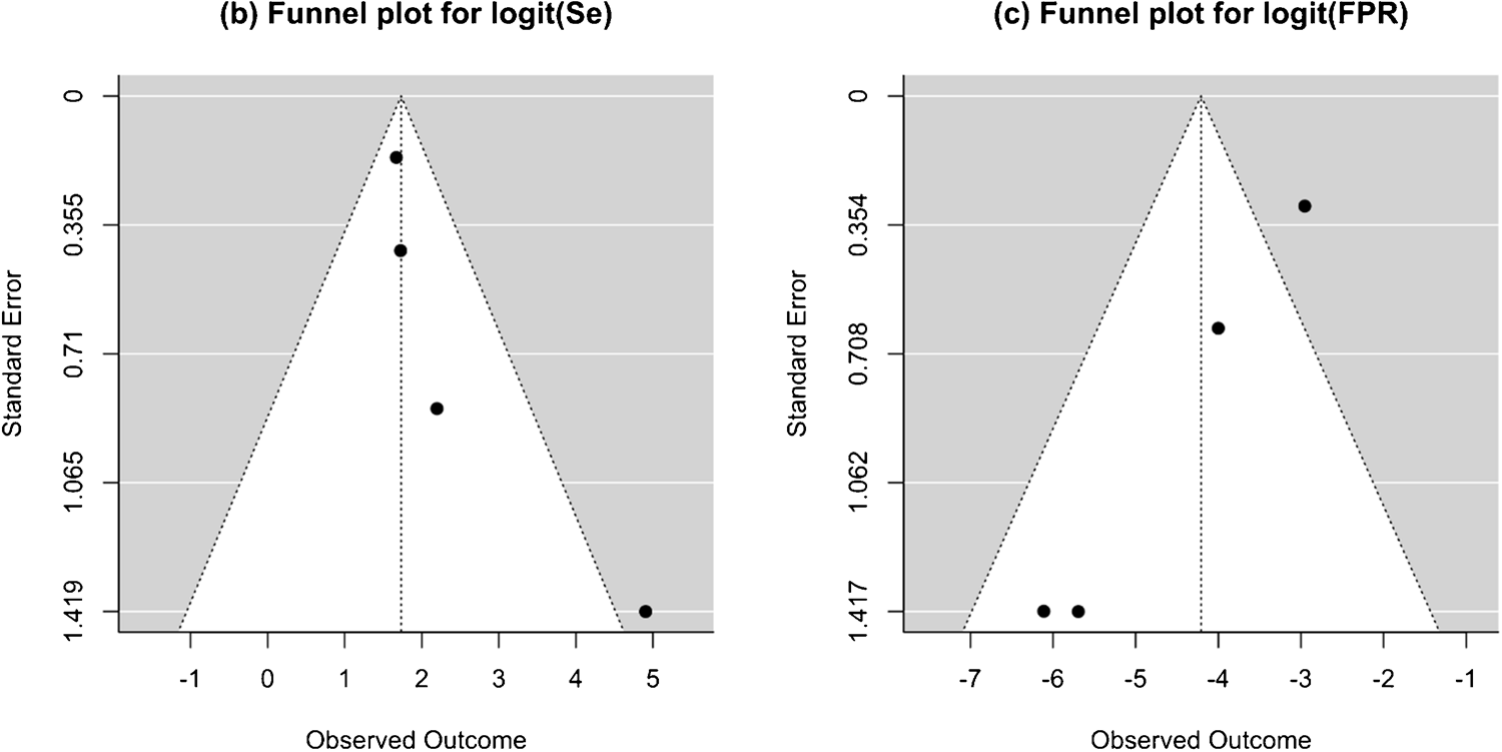
Funnel plots for the assessment of the effect of potential publication bias/small study effect in the reported diagnostic performance indices in overall meta-analysis. FPR, false positive rate; Sen, sensitivity

Figure 2 displays the paired funnel plots associated with the overall meta-analysis to assess the potential effects of publication bias or small study effects. Although no statistical test was conducted to specifically evaluate publication bias, a visual examination of these plots reveals evidence of asymmetry despite the limited number of studies.

### Meta-analysis

**Fig. 3:**
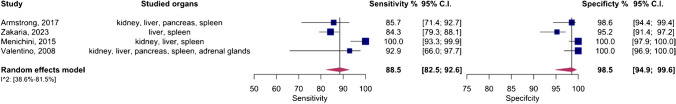
Paired forest plots of the bivariate model random effect meta-analysis of diagnostic performance of contrast-enhanced sonography (CEUS) in diagnosing various solid organ injuries in pediatric patients (overall analysis). CI, confidence interval

**Fig. 4:**
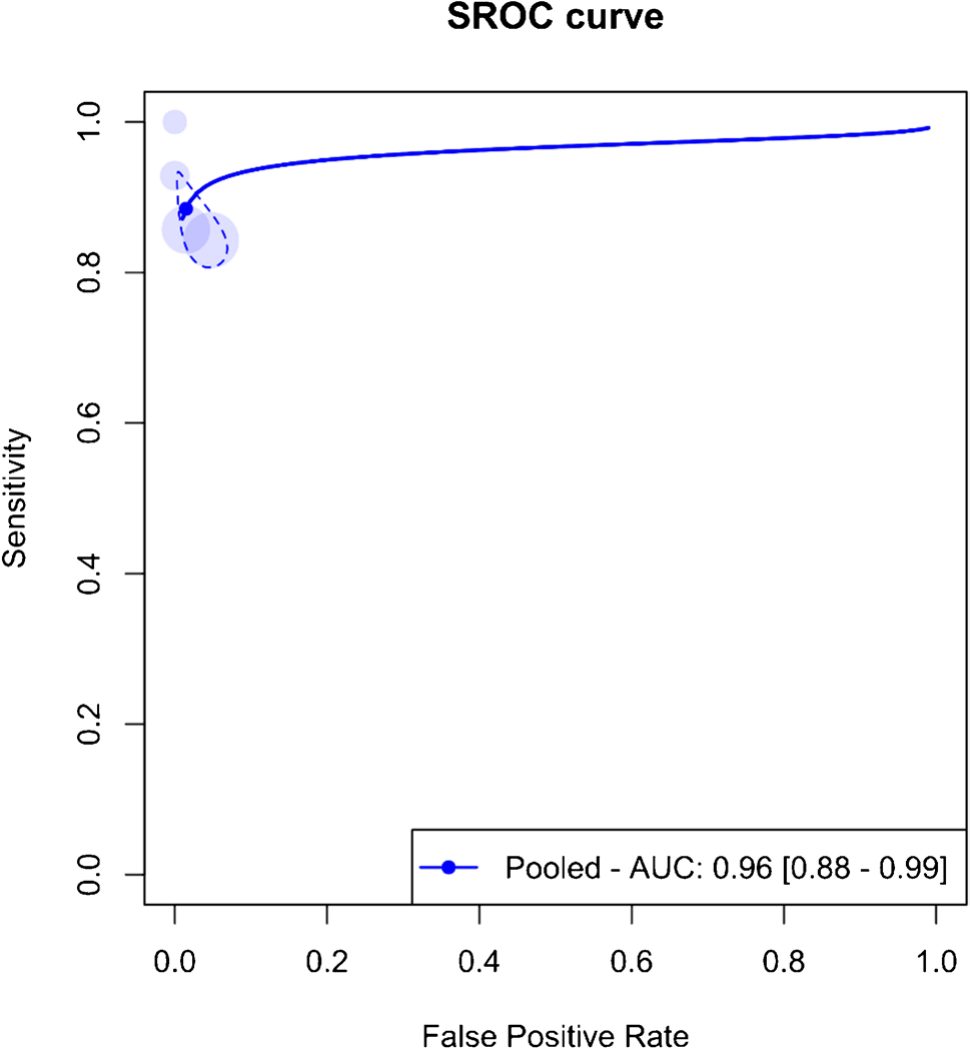
Summary receiver operating curve (SROC) of the bivariate model random effect meta-analysis of diagnostic performance of contrast-enhanced sonography (CEUS) in diagnosing various solid organ injuries in pediatric patients (overall analysis). AUC, area under the curve; SROC, summary receiver operating curve

An overall meta-analysis was initially conducted, pooling data regardless of the specific organs involved. Figure 3 displays the paired forest plots for this comprehensive analysis, revealing a pooled sensitivity of 88.5% (95%CI 82.5–92.6%) and a pooled specificity of 98.5% (95%CI 94.9–99.6%). Additionally, Fig. 4 illustrates the SROC curve for the entire meta-analysis, indicating an AUC of 96% (95%CI 88–99%).

Significant heterogeneity (*I*^2^ = 95%, 95%CI 38.6–81.5%) was observed across the four studies, prompting sensitivity analysis. This analysis identified the study by Menichini et al. as an outlier. The pooled sensitivity and specificity post-exclusion of this study were 84.8% (95%CI 80.4–88.4%) and 96.9% (95%CI 93.6–98.6%), respectively, with an AUC of 89% (95%CI 83–99%).

**Fig. 5:**
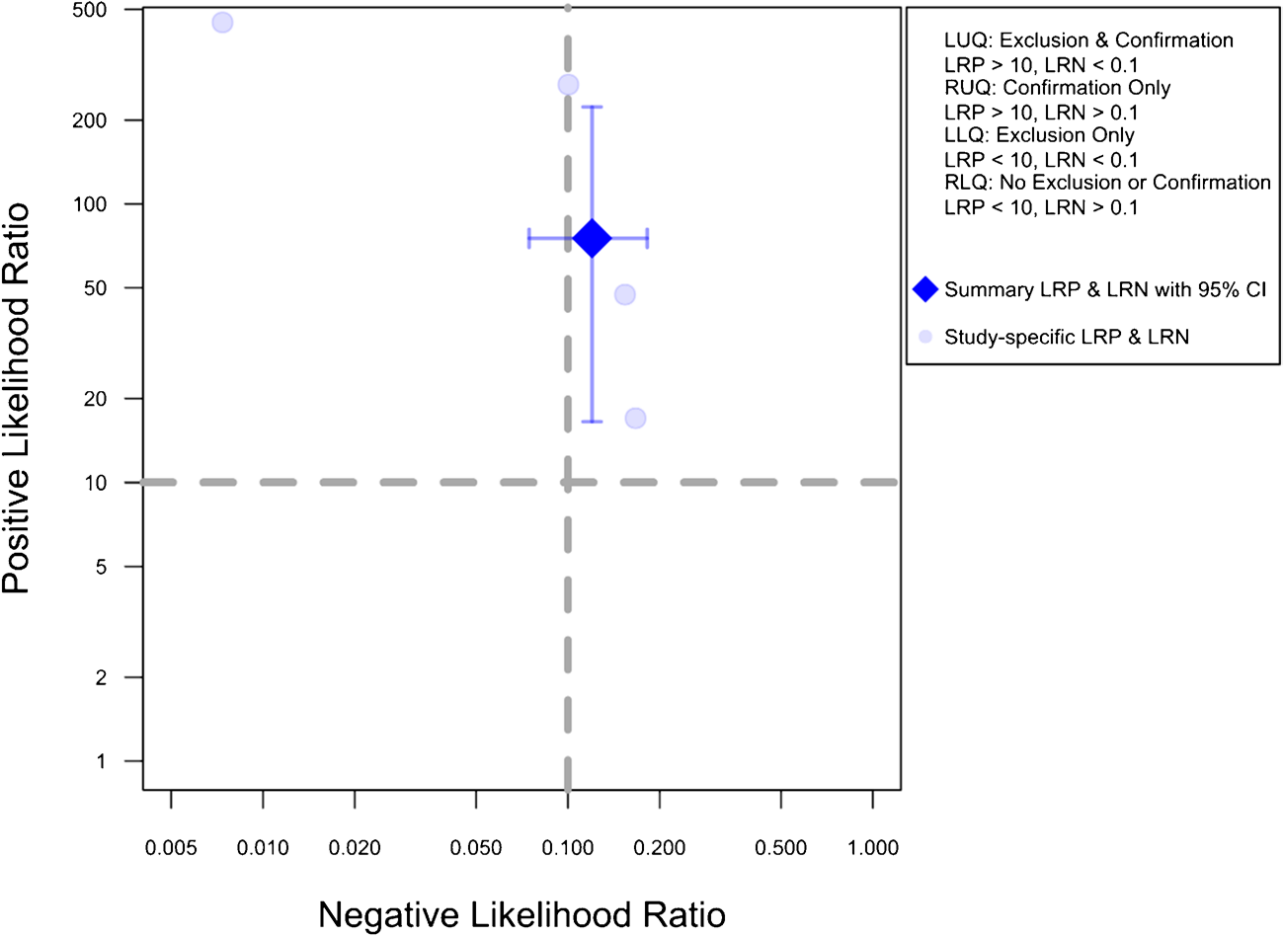
Likelihood scattergram of the bivariate model random effect meta-analysis of diagnostic performance of contrast-enhanced sonography (CEUS) in diagnosing various solid organ injuries in pediatric patients (overall analysis). The scattergram is divided into four quadrants based on likelihood ratios, with data points having a positive likelihood ratio (LRP) greater than 10 classified as optimal for confirmation, and those with a negative likelihood ratio (LRN) less than 0.1 classified as optimal for exclusion. LLQ, left lower quadrant; LRN, negative likelihood ratio; LRP, positive likelihood ratio; LUQ, left upper quadrant; RLQ, right lower quadrant; RUQ, right upper quadrant

**Fig. 6:**
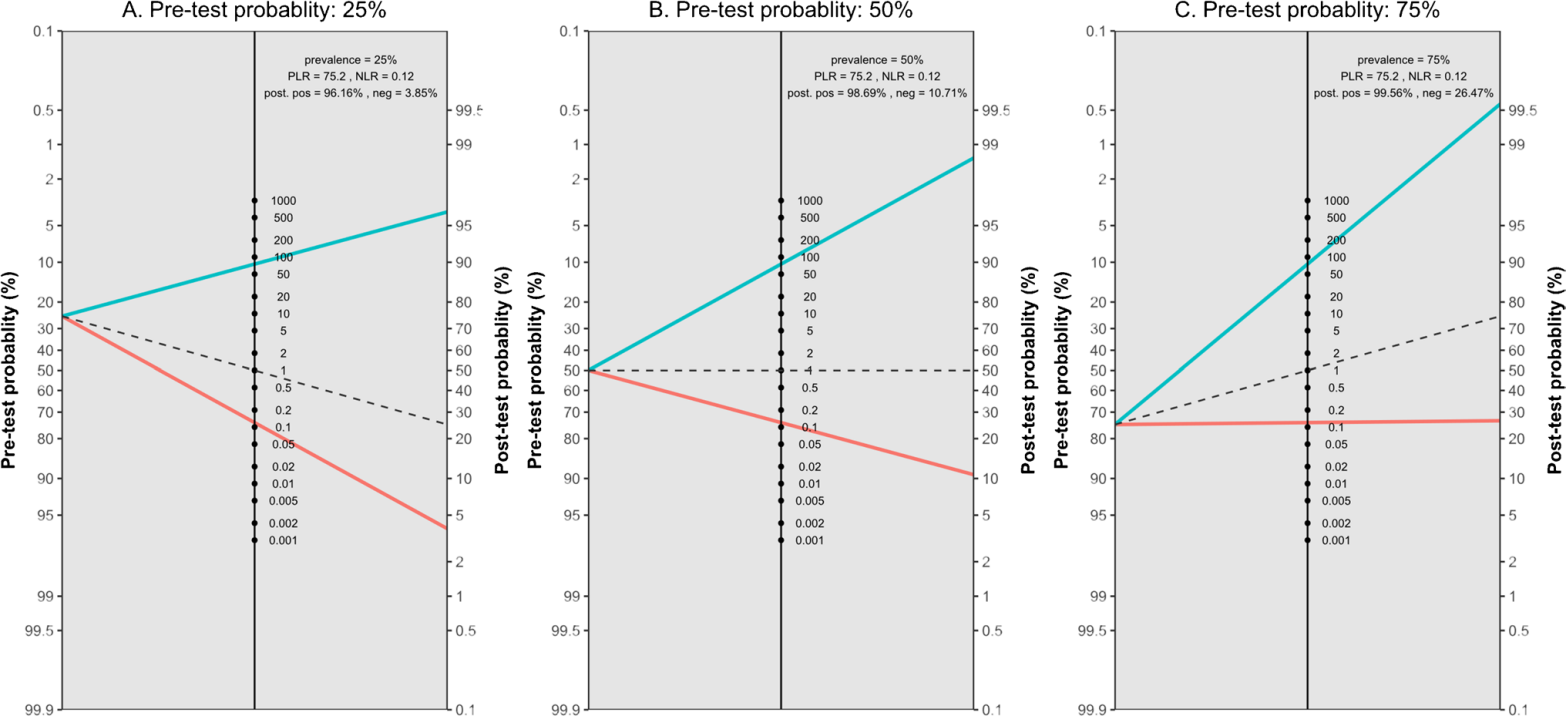
Fagan’s nomogram plots with three different pre-test probability assumptions and pooled diagnostic performance indices based on the bivariate model random effect meta-analysis of diagnostic performance of contrast-enhanced sonography (CEUS) in diagnosing various solid organ injuries in pediatric patients (overall analysis). Neg, negative; NLR, negative likelihood ratio; PLR, positive likelihood ratio; Pos, positive

Figure 5 presents the likelihood ratio scattergram for this general meta-analysis. The observations in likelihood ratio values are supported by Fagan’s nomogram, as presented in Fig. 6, indicating that the post-test probability of a solid organ injury remains above 95%, even with a low pre-test probability of 25%. Conversely, in scenarios with a high pre-test probability of 75%, CEUS may still miss 26% of solid organ injuries. Notably, these findings are largely consistent even after the exclusion of the outlier study by Menichini et al., and the post-test probability remained higher than 90% (90.9%) even with a pre-test probability as low as 25%, whereas the post-test probability after a negative test with a pre-test probability of 75% was calculated as 29.8%.

### Subgroup analysis

**Fig. 7:**
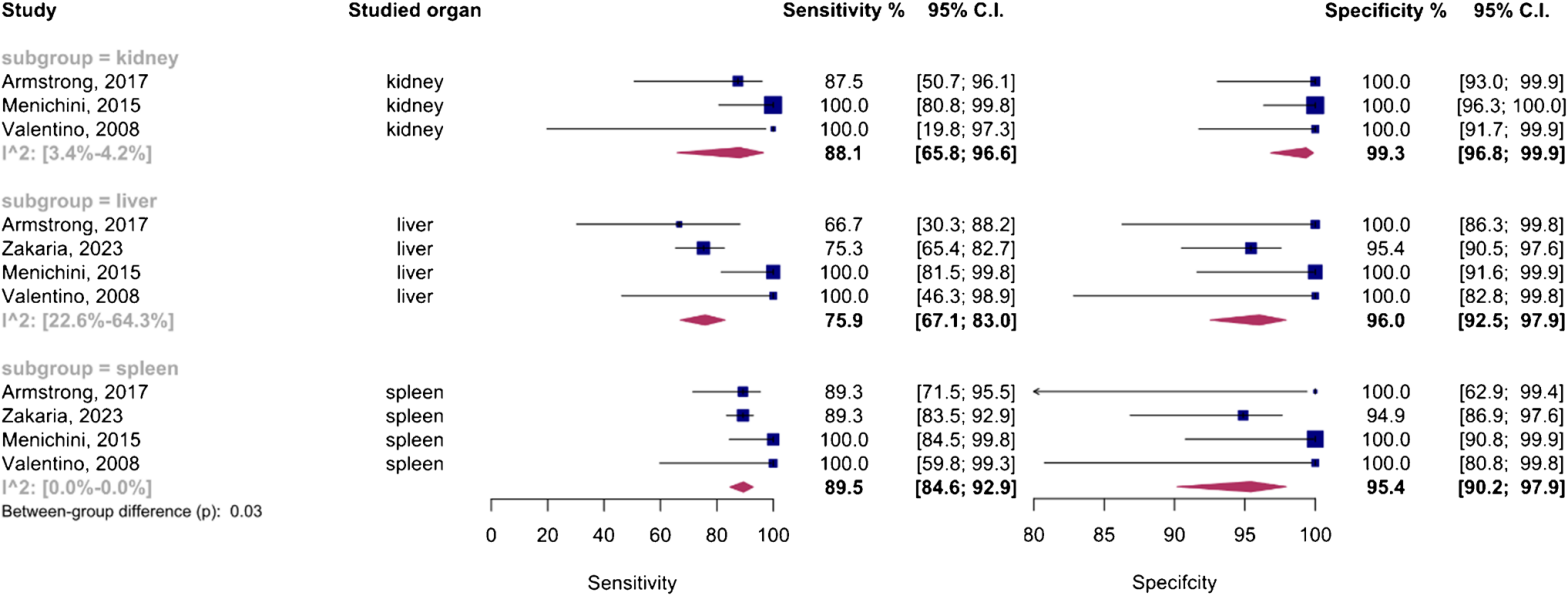
Paired forest plots of the bivariate model random effect meta-analysis of diagnostic performance of contrast-enhanced sonography (CEUS) in diagnosing solid organ injuries of the kidney, liver, and spleen in pediatric patients (subgroup analysis). CI, confidence interval

**Fig. 8:**
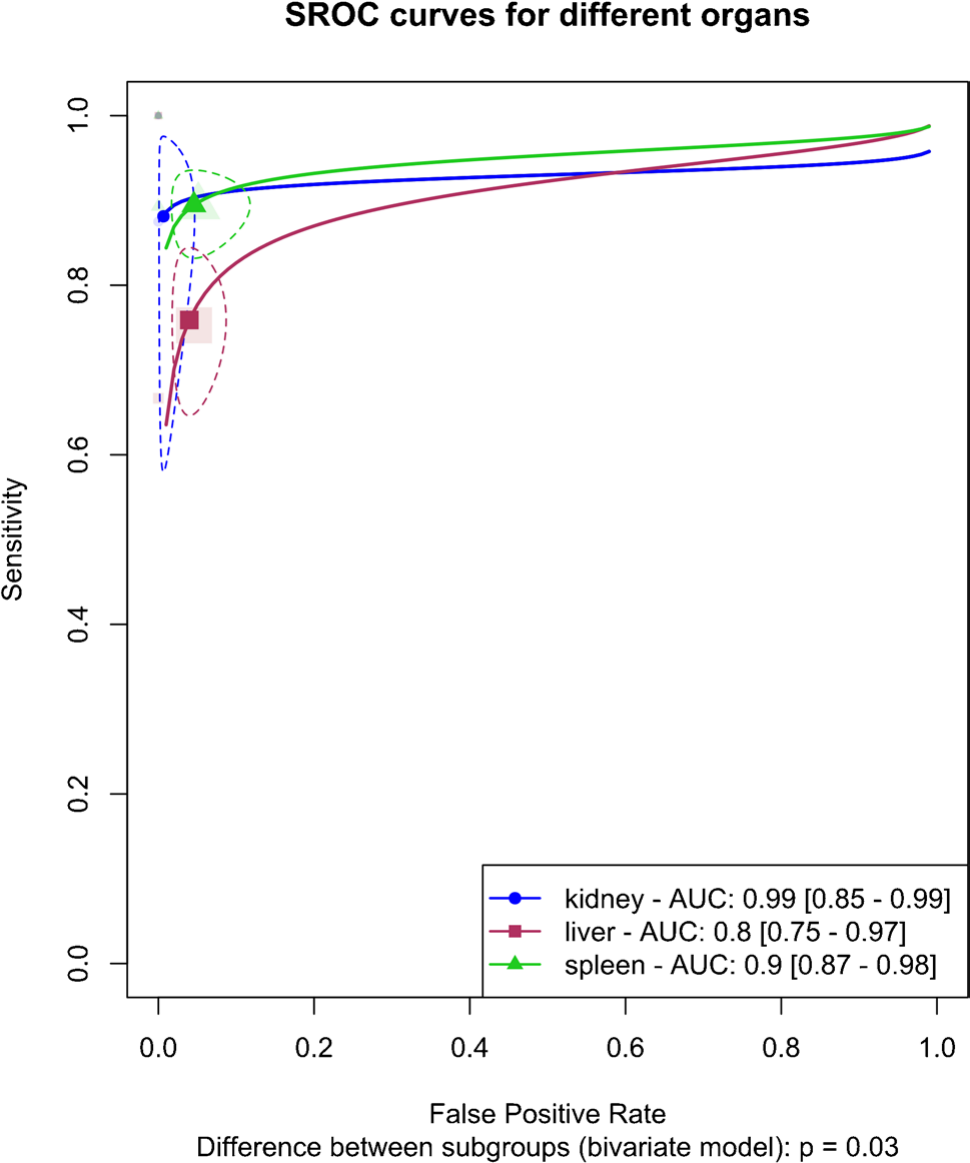
Summary receiver operating curves (SROCs) of the bivariate model random effect meta-analysis of diagnostic performance of contrast-enhanced sonography (CEUS) in diagnosing solid organ injuries of the kidney, liver, and spleen in pediatric patients (subgroup analysis). AUC, area under the curve; SROC, summary receiver operating curve

The analysis included an organ-specific subgroup assessment focusing on kidney, liver, and spleen injuries, each represented by at least three studies, as illustrated in Fig. 7. The pooled sensitivities for these subgroups were 88.1% (95%CI 65.8–96.6%) for the kidney, 75.9% (95%CI 67.1–83%) for the liver, and 89.5% (95%CI 84.6–92.9%) for the spleen. The corresponding pooled specificities were 99.3% (95%CI 96.8–99.9%) for the kidney, 96% (95%CI 92.5–97.9%) for the liver, and 95.4% (95%CI 90.2–97.9%) for the spleen. Figure 8 presents the summary receiver operating characteristic (SROC) curves for these subgroups, with pooled AUCs of 99% (95%CI 85–99%) for the kidney, 80% (95%CI 75–97%) for the liver, and 90% (95%CI 87–98%) for splenic injuries. Significant differences were observed between subgroups (*P*-value = 0.03), with post hoc analysis indicating higher specificity in the kidney compared to other organs (*P*-value < 0.05) and lower sensitivity in liver injuries compared to splenic injuries (*P*-value < 0.01).

The liver subgroup demonstrated significant heterogeneity (*I*^2^ 95%CI 22.6–64.3%), leading to a sensitivity analysis that identified Menichini et al.’s study as an outlier. Importantly, subgroup analysis after removing this outlier showed that the higher sensitivity in the spleen compared to the liver remained significant (*P*-value < 0.01), whereas the higher specificity in renal injuries was no longer significant (*P*-value = 0.09).

**Fig. 9:**
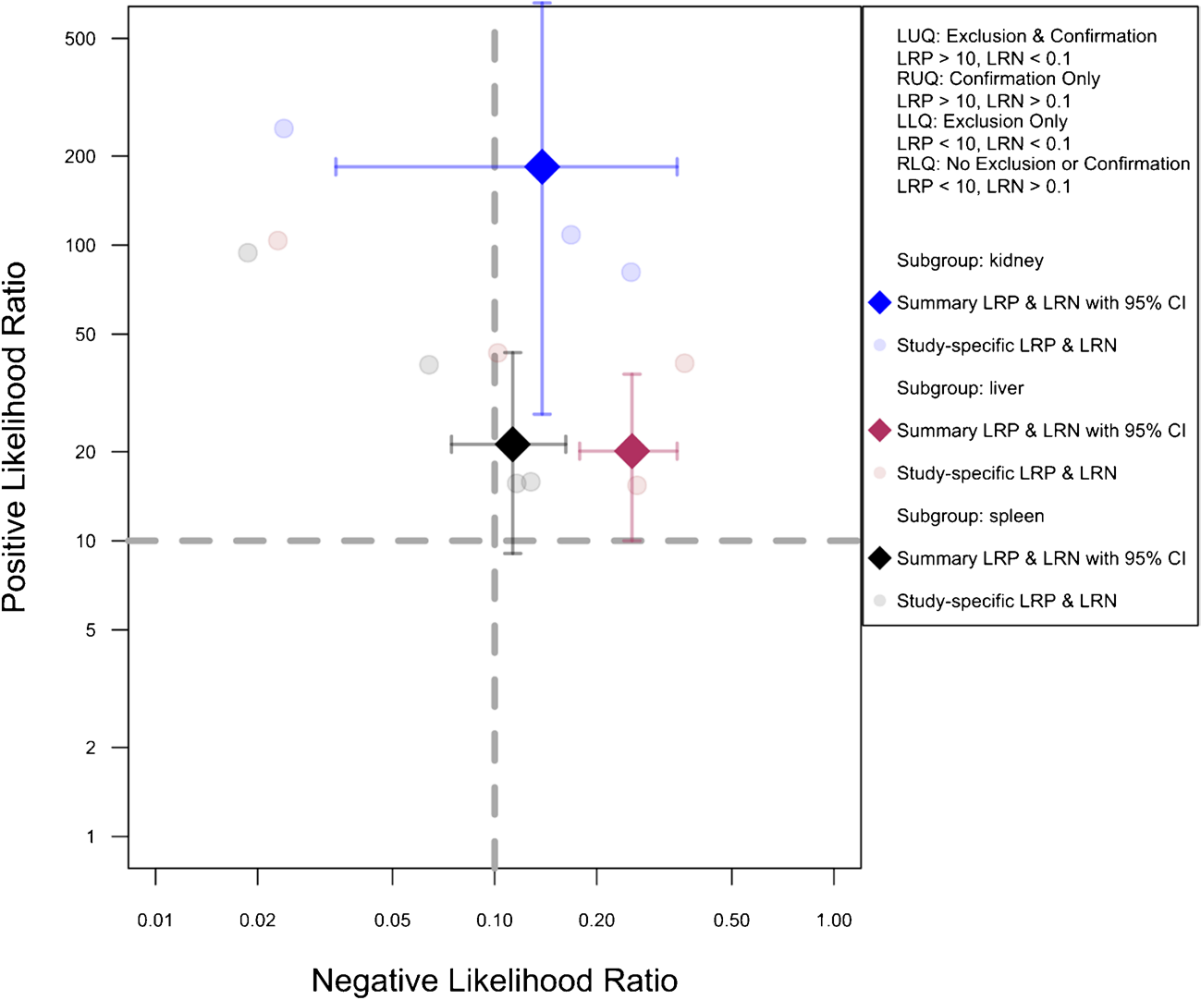
Likelihood scattergram of the bivariate model random effect meta-analysis of diagnostic performance of contrast-enhanced sonography (CEUS) in diagnosing solid organ injuries of the kidney, liver, and spleen in pediatric patients (subgroup analysis). The scattergram is divided into four quadrants based on likelihood ratios, with data points having a positive likelihood ratio (LRP) greater than 10 classified as optimal for confirmation, and those with a negative likelihood ratio (LRN) less than 0.1 classified as optimal for exclusion. CI, confidence interval; LLQ, left lower quadrant; LRN, negative likelihood ratio; LRP, positive likelihood ratio; LUQ, left upper quadrant; RLQ, right lower quadrant; RUQ, right upper quadrant

Figure 9 illustrates the pre-exclusion likelihood ratio scattergrams for this analysis. Supplementary Figs. 1, 2, and 3 present the pre-exclusion Fagan’s nomogram plots for the kidney, liver, and spleen, respectively, with a post-test probability exceeding 40% in cases of high suspicion (pre-test 75%) but negative CEUS results.

## Discussion

Pediatric patients are generally considered well-suited candidates for diagnostic ultrasound due to inherent advantages such as smaller levels of abdominal fat tissue and smaller body size, which allows for better visualization of imaging findings [34–36]. Ultrasound is also often the first-line imaging modality for many indications in pediatric abdominal imaging due, in part, to its lack of ionizing radiation [37]. Despite these benefits, the use of ultrasound in pediatric blunt abdominal trauma has been limited by lower diagnostic accuracy, particularly in sensitivity and false-negative rates, for solid organ injury [38]. Studies suggest that diagnostic ultrasound may miss up to a third of solid organ injuries visible on CT scans in children [11, 39]. Attempts to enhance diagnostic ultrasound’s accuracy in detecting free fluid and parenchymal injuries through methods such as tissue harmonic imaging, Doppler techniques, and high-resolution transducers have largely failed to achieve satisfactory detection rates of solid organ [40–42].

As such, the current systematic review and meta-analysis synthesized the results of four original studies that investigated the diagnostic performance of CEUS in assessing pediatric solid organ injuries caused by BAT, using CT as a reference standard. The main analysis demonstrated consistently high levels of diagnostic accuracy, with a pooled sensitivity of 88.5% (95%CI 82.5–92.6%), specificity of 98.5% (95%CI 94.9–99.6%), and an AUC of 96% (95%CI 88–99%). In addition, a novel finding from our subgroup meta-analysis was the lower sensitivity in detecting hepatic injuries compared to splenic injuries (*P*-value < 0.01). This difference could be attributed to the challenging accessibility of certain liver parenchyma regions, especially the dome and lateral segments, during examinations, particularly in less cooperative children [43].

The findings of the present study are consistent with those of Pegoraro et al., who reported CEUS to have a sensitivity ranging from 85.7% to 100% and a specificity between 89 and 100% for detecting solid organ injuries in pediatric BAT cases [22]. Their review also emphasized the safety and accuracy of CEUS while noting the need for further research to assess its feasibility when performed by non-radiologists, as well as to evaluate the necessary training and cost-effectiveness in reducing CT scan use [22]. Similarly, Zhang et al.’s meta-analysis demonstrated the high diagnostic potential of CEUS in BAT patients across all age groups, reporting a pooled AUC of 0.98, which reflects excellent diagnostic accuracy [21]. Another recent meta-analysis comparing CEUS with diagnostic ultrasound in abdominal trauma cases in children and adults using CT as a reference standard confirmed CEUS’s superiority over diagnostic ultrasound, showing pooled sensitivity and specificity rates of 93.3% and 99.5%, respectively, for detecting abdominal solid organ injuries [44]. Although our meta-analysis supports these findings, we observed slightly lower sensitivity, possibly due to age-related differences in the study populations, which highlights the need for additional research to explore factors contributing to this discrepancy.

Notably, Fagan nomograms and likelihood ratio scattergrams in our study revealed that while CEUS is effective for confirming solid organ injuries, it has limitations in excluding suspected injuries, in both patients with high and low pre-test probability. The post-test probability of injury remained elevated even in cases with negative CEUS results. Notably, for liver injuries, the post-test probability exceeded 40%, even with a high pre-test probability of 75% and a negative CEUS result. Additionally, the positive likelihood ratio of less than 10 in our analysis suggests CEUS’s limited ability to exclude lesions despite its strong performance in confirming injuries in the liver, spleen, and kidneys. This aligns with Menichini et al.’s conclusion that while the diagnostic accuracy of CEUS approaches that of CT, it may not be suitable for ruling out injuries due to its slightly lower sensitivity and the risk of missing certain injuries [20]. Given this, CEUS may be a viable method for serial examinations and follow-up imaging [20, 45]. While not all pediatric blunt abdominal trauma cases require follow-up imaging, specific indications exist, such as in patients with minor splenic or hepatic trauma managed conservatively.

While CEUS is effective for detecting renal parenchymal injuries, it is not suitable for identifying injuries to the renal collecting system due to the lack of urinary excretion of ultrasound-based contrast agents and may under-stage renal trauma [46]. For instance, the study by Menichini et al. reported that although CEUS did not miss any parenchymal lesions, it failed to detect both cases of urinomas in their cohort [20]. Therefore, in cases where there is any suspicion of urinary tract injury, CE-CT should be performed to ensure accurate diagnosis and staging [47]. The diagnostic efficacy of CEUS is also limited in cases of mesenteric injuries, bowel injuries, diaphragmatic ruptures, and retroperitoneal injuries, including adrenal and pancreatic lesions, due to suboptimal visualization. This necessitates CT evaluation in suspected cases of these injuries [43].

Managing pain in pediatric trauma patients is crucial in emergency departments, as inadequate pain control not only compromises the quality of medical examinations but also increases anxiety and fear of healthcare providers among children [48]. One significant but often overlooked issue is the discomfort caused by the prolonged placement of ultrasound probes in emergency settings. Although some studies on pediatric trauma patients with various injuries have assessed pain levels and generally reported non-significant findings, there is a notable gap in research concerning pain assessment in CEUS for solid organ injuries [49, 50]. This gap is particularly concerning given that the number of patients for whom CEUS could not be performed due to abdominal pain was not specified in the reviewed studies. The absence of this data represents a critical oversight, as it could introduce bias into the results. Therefore, future studies are needed not only to evaluate the pain levels and potential psychological impact of extended ultrasound probe use in pediatric trauma care settings but also to ensure comprehensive reporting on cases where CEUS is not performed due to pain.

A key limitation of this meta-analysis is the small sample sizes of the included studies, which may reduce the statistical power of our findings. The limited number of studies, combined with small individual sample sizes, increases the likelihood of sampling and selection biases. Additionally, there was significant heterogeneity among the studies, particularly in terms of population characteristics, examination methods, and workflow, which may affect the generalizability of the results. Notably, the study by Menichini et al. stood out as a significant outlier in both the overall and organ-specific subgroup analyses, reporting unusually high diagnostic accuracy for CEUS [20]. This anomaly is likely due to the selective inclusion of patients with minor trauma and at least one suspicious finding on diagnostic ultrasound, introducing both sampling and confirmation bias [20]. These factors further emphasize the need for caution when interpreting the findings. Finally, the assessment of publication bias raised concerns about potential small-study effects and publication bias, which could also influence the results.

A critical consideration of the present study is that it has demonstrated that CEUS is more suited as a confirmatory tool rather than for excluding injuries. Given this, discussing a strategy that compares CT and CEUS as alternative diagnostic methods may be premature. Further research is needed to clarify CEUS’s role in pediatric trauma, particularly in terms of its use as a complementary tool to CT rather than as a replacement.

## Conclusion

Contrast-enhanced ultrasound has high sensitivity and specificity in evaluating pediatric solid organ injuries resulting from BAT. Additionally, analysis of pooled likelihood ratios emphasizes its utility in confirming the presence of solid organ injuries in suspected cases, although it demonstrates suboptimal efficacy in excluding such injuries. Additional comprehensive large-scale studies are needed to confirm its diagnostic accuracy and subsequently define best practice guidelines for CEUS in pediatric trauma care.

## Supplementary Information

Below is the link to the electronic supplementary material.

Supplementary file1 (DOCX 1134 KB)

## Data Availability

Data supporting the findings of this study are available within the paper. Further details of the extracted data from the included studies are available from the corresponding author upon reasonable request.

## Declarations

### Generative AI and AI-assisted technologies in the writing process

We acknowledge ChatGPT, an OpenAI language model based on the GPT-4 architecture, for assisting with language corrections during the article’s editing. The model enhanced the readability and language quality of the publication. However, the authors retain full responsibility for the content, having reviewed and edited it as needed after using the tool.
