# Supplementary material for: Diagnostic performance of contrast-enhanced ultrasound in traumatic solid organ injuries in children: a systematic review and meta-analysis
Source: Pediatr Radiol. 2024 Dec 13;55(2):226–41. doi: 10.1007/s00247-024-06127-9 (PMC11805793; doi:10.1007/s00247-024-06127-9)
Supplement: Supplementary file 1 — Supplementary file1 (DOCX 1134 KB) [file 247_2024_6127_MOESM1_ESM.docx]

**Article title:**

Diagnostic performance of contrast‑enhanced ultrasound in traumatic solid organ injuries in children

**Journal name:**

Pediatric Radiology


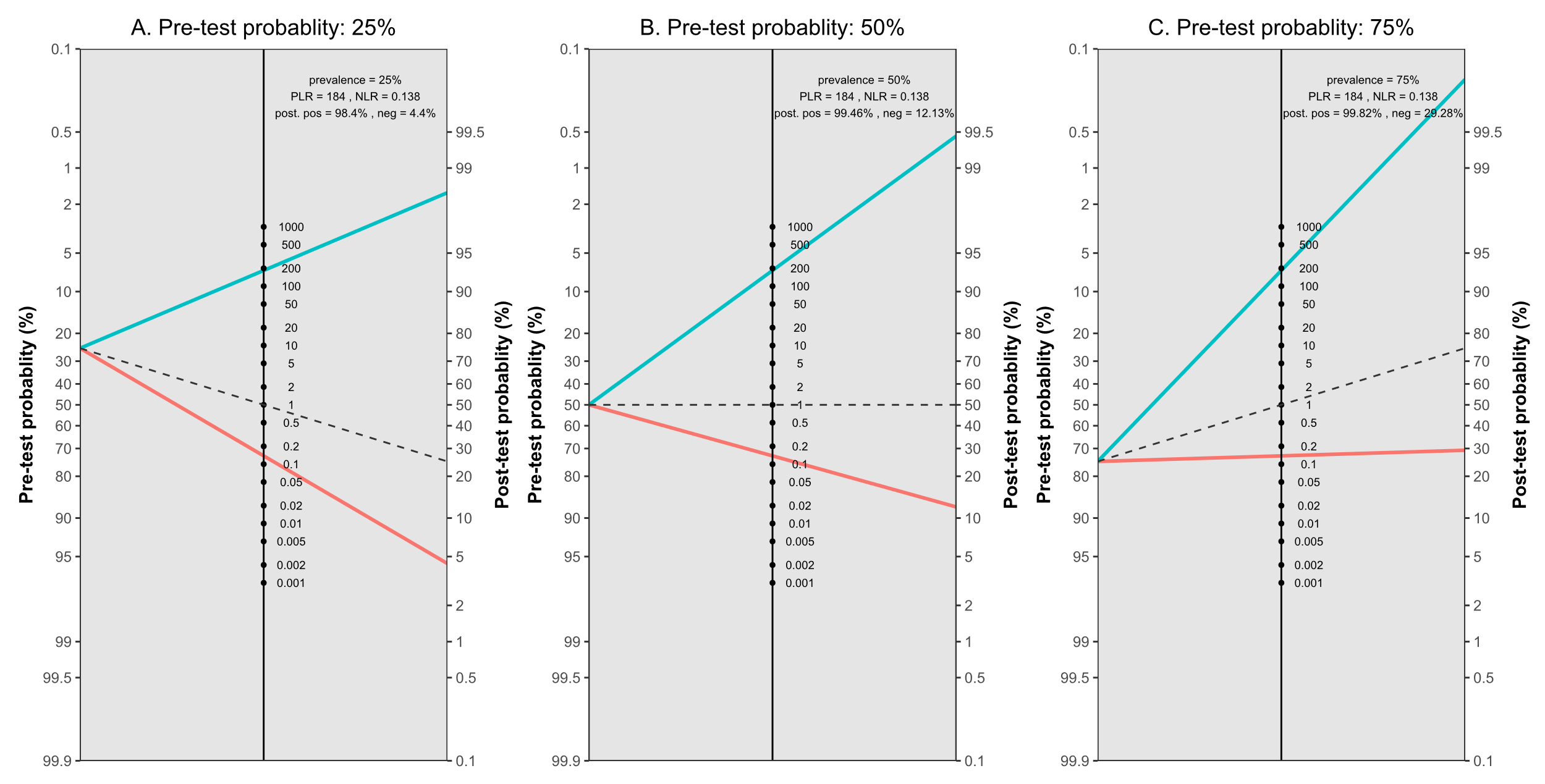


**Supplementary Fig. 1** Fagan's nomogram plots with three different pre-test probability assumptions and pooled diagnostic performance indices based on the bivariate model random effect meta-analysis of diagnostic performance of contrast-enhanced sonography (CEUS) in diagnosing kidney injuries in pediatric patients; Neg: Negative; NLR: negative likelihood ratio; PLR: Positive likelihood ratio; Pos: Positive


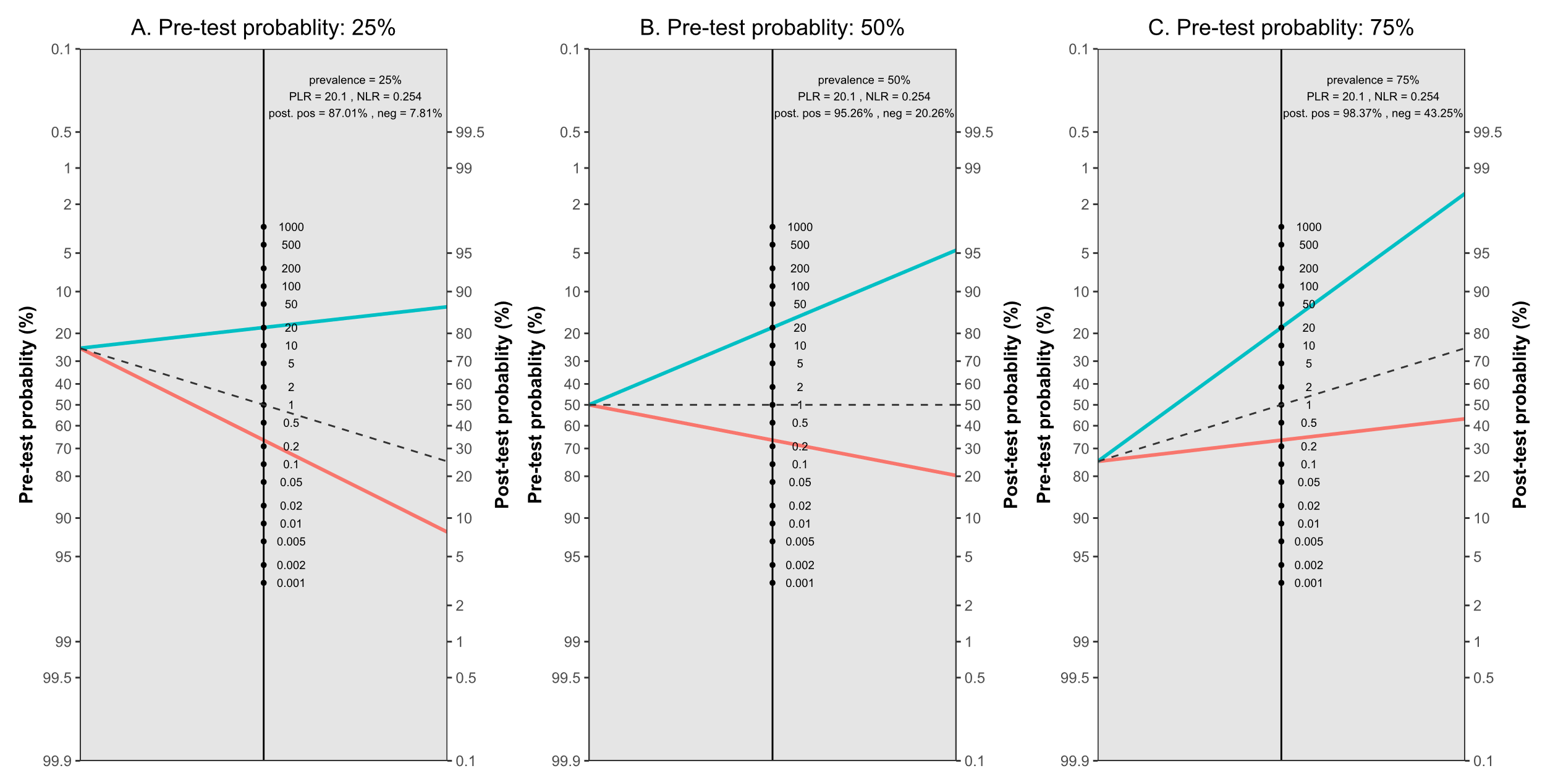


**Supplementary Fig. 2** Fagan's nomogram plots with three different pre-test probability assumptions and pooled diagnostic performance indices based on the bivariate model random-effect meta-analysis of diagnostic performance of contrast-enhanced sonography (CEUS) in diagnosing liver injuries in pediatric patients; Neg: Negative; NLR: Negative likelihood ratio; PLR: Positive likelihood ratio; Pos: Positive


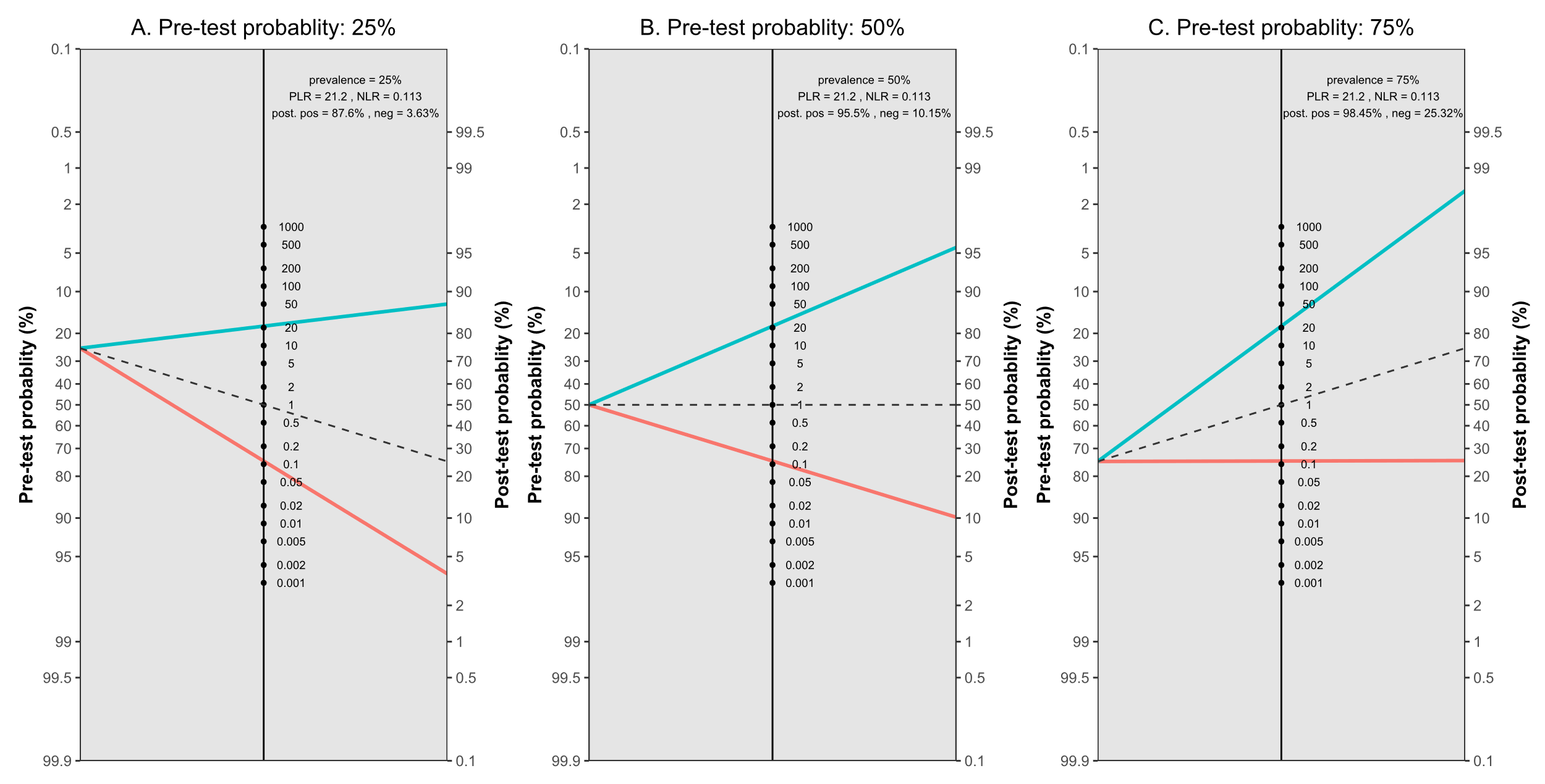


**Supplementary Fig. 3** Fagan's nomogram plots with three different pre-test probability assumptions and pooled diagnostic performance indices based on the bivariate model random-effect meta-analysis of diagnostic performance of contrast-enhanced sonography (CEUS) in diagnosing spleen injuries in pediatric patients; Neg: Negative; NLR: Negative likelihood ratio; PLR: Positive likelihood ratio; Pos: Positive
